# Whole exome sequencing in Japanese spinocerebellar ataxia identifies novel variants

**DOI:** 10.1038/s10038-025-01405-2

**Published:** 2025-09-12

**Authors:** Tomoaki Watanabe, Kodai Kume, Ken Inoue, Masataka Nakamura, Shinji Yamamoto, Takashi Kurashige, Tomohiko Ohshita, Taku Tazuma, Misako Kaido, Yuta Maetani, Hirofumi Maruyama, Hideshi Kawakami

**Affiliations:** 1 Department of Molecular Epidemiology, Research Institute for Radiation Biology and Medicine, Hiroshima University, Hiroshima, Japan; 2 Department of Clinical Neuroscience and Therapeutics, Graduate School of Biomedical and Health Sciences, Hiroshima University, Hiroshima, Japan; 3 Inoue Neurology Clinic, Hiroshima, Japan; 4 Department of Neurology, Kansai Medical University, Osaka, Japan; 5 Department of Neurology, Hyogo Prefectural Rehabilitation Hospital, Nishi-harima, Tatsuno, Japan; 6 Department of Neurology, NHO Kure Medical Center, and Chugoku Cancer Center, Kure, Japan; 7 Department of Neurology, Sakai City Medical Center, Osaka, Japan

**Keywords:** Neurodegenerative diseases, Medical genetics

## Abstract

Spinocerebellar degeneration (SCD) is a clinically and genetically diverse group, and the dominant form of SCD (AD-SCD) is generally referred to as spinocerebellar ataxia (SCA) that primarily affects the cerebellum. Some patients do not have a definitive genetic diagnosis but may carry unknown variants of known causative genes. Here, we screened for known SCA-associated genes in patients with suspected SCA. We examined 174 patients with SCA lacking abnormal repeat expansion of known causative genes. Whole-exome sequencing (WES) was performed to screen for variants in SCA-associated genes. The identified variants were confirmed by Sanger sequencing, and their pathogenicity was determined using five web-based algorithms. WES revealed novel single-nucleotide variants (SNVs) in three genes, *ELOVL4*, *ELOVL5*, and *GRM1*. Patients presented with symptoms other than cerebellar symptoms. One patient with an *ELOVL4* variant exhibited skin changes, a typical symptom of *ELOVL4* SCA, whereas the other *ELOVL4* SCA patient had no skin changes and exhibited mild parkinsonism and calcification in the globus pallidus and dentate nucleus. The patient with an *ELOVL5* variant exhibited bladder and rectal disturbances. Finally, patients with *GRM1* variants showed few common features beyond the cerebellar symptoms. One patient showed white matter lesions, cognitive decline, and no-no head tremors, whereas the other showed spasticity. The identification of novel SNVs in these known SCA-associated genes will expand our understanding of the genetic landscape of SCA and facilitate the diagnosis of previously undiagnosed patients.

## Introduction

Spinocerebellar degeneration (SCD) is a clinically and genetically diverse group, and the dominant form of SCD (AD-SCD) is generally referred to as spinocerebellar ataxia (SCA). SCA primarily affects the cerebellum and its associated pathways. SCA is clinically characterized by progressive ataxia, dysarthria, and oculomotor disturbances. Numerous novel genes have been identified by next-generation sequencing techniques [1]. More than 50 genes are listed as causative genes of SCA in Online Mendelian Inheritance in Man (OMIM, https://www.omim.org/). The large number of pathogenic variants of SCA highlights the complexity and diversity of its molecular etiology. Despite significant progress in the identification of disease-causing mutations, some patients do not have a definitive genetic diagnosis.

Whole exome sequencing (WES) enables the comprehensive screening of coding regions across the genome, facilitating the detection of both novel and previously reported single-nucleotide variants (SNVs) that underlie inherited disorders. Although WES has some limitations [2], it is a powerful tool for uncovering the genetic basis of diseases, including ataxia [3, 4].

In this study, we aimed to perform WES on a cohort of patients presenting with clinical features consistent with SCA. Our analysis revealed several novel SNVs in genes previously associated with the disorder. Specifically, we identified novel variants of *ELOVL4*, *ELOVL5*, and *GRM1*. The identification of novel SNVs in these known SCA-causing genes can expand our understanding of the genetic landscape of SCA.

## Materials and methods

### Subjects

We examined 174 patients with SCA who had no abnormal repeat expansions of known causative genes (for SCA1, SCA2, SCA3, SCA6, SCA7, SCA8, SCA31, SCA36, and dentatorubral–pallidoluysian atrophy). Each participant was diagnosed with SCA by neurologists. The mean age of the participants was 46.3 years (SD = 16.1, range = 10–82). Dominant inheritance was suspected in 173 cases out of 174 cases, and only one patient had no family history but was highly suspected of having SCA based on symptoms. The Ethics Committee of Hiroshima University approved the study protocol. All experiments were conducted after obtaining written informed consent from the patients or their family members.

### Genetic analysis

Genomic DNA was isolated from the peripheral blood leukocytes of the patient using QuickGene-610L (Wako, Osaka, Japan). WES was performed using the BGI platform (Shenzhen, Guangdong, China). Mapping to the human reference genome (GRCh38) was performed using the BWA tool, with the removal of duplicate reads using Picard. Variant calls and annotations were made using GATK and Annovar software. We analyzed the variants of known SCA genes including *SPTBN2*, *TTBK2*, *KCNC3*, *PRKCG*, *ITPR1*, *KCND3*, *TMEM240*, *PDYN*, *PNPT1*, *EEF2*, *FGF14*, *AFG3L2*, *ITPR1*, *ELOVL4*, *TGM6*, *ELOVL5*, *CCDC88C*, *TRPC3*, *CACNA1G*, *MME*, *GRM1*, *FAT2*, *PLD3*, *STUB1*, *SAMD9L*, and *NPTX1*. We included the variants as candidate using the criteria: frequency in the database < 0.001; missense, nonsense, and indel variants; and depth > 4. The candidate variants were verified using Sanger sequencing using an Applied Biosystems 3130 DNA Sequencer (Life Technologies). Variants were evaluated by predictive algorithms (AlphaMissense [https://alphamissense.hegelab.org/], Combined Annotation Dependent Depletion [CADD, https://cadd.gs.washington.edu/], Sorting Intolerant From Tolerant [SIFT, https://sift.bii.a-star.edu.sg/], PolyPhen-2 [http://genetics.bwh.harvard.edu/pph2/], and MutationTaster [http://www.mutationtaster.org/]), which predicted whether amino acid substitutions would affect protein function. Allele frequencies were obtained from gnomAD (https://gnomad.broadinstitute.org/) and the National Center Biobank Network (NCBN) database [5] using TogoVar (https://grch38.togovar.org/).

## Results

### Overview

WES of the cohort of patients with clinical symptoms consistent with SCA revealed novel SNVs in *ELOVL4*, *ELOVL5*, and *GRM1* in 5 patients. The genomic and clinical features of each patient are summarized in Table 1. We also identified 49 benign variants in 42 individuals and no known pathogenic variants in SCA-associated genes. The diagnostic yield was 2.9% (5/174).

**Table 1:** Genomic and clinical features of the patients

Patient number	1	2	3	4	5
Genomic features					
Gene	*ELOVL4*	*ELOVL4*	*ELOVL5*	*GRM1*	*GRM1*
Chromosome	6	6	6	6	6
Position	79921659	79919547	53291843	146434774	146398888
Reference base	C	G	G	A	T
Alternative base	G	A	A	C	C
cytoBand	6q14.1	6q14.1	6p12.1	6q24.3	6q24.3
Exonic Function	Nonsynonimous	Nonsynonimous	Nonsynonimous	Nonsynonimous	Nonsynonimous
Amino Acid change	NM_022726 Exon 4 c.507 G > C p.W169C	NM_022726 Exon 5 c.542 C > T p.A181V	NM_001242828 Exon 3 c.179 C > T p.S60F	NM_001278064 Exon 8 c.3563 A > C p.K1188T	NM_001278064 Exon 7 c.1849T>C p.Y617H
AlphaMissense	**Likely pathogenic (0.964)**	**Likely pathogenic (0.886)**	Ambiguous (0.470)	Likely Benign (0.191)	Likely Benign (0.247)
CADD	**28.6**	24.9	25.1	22.5	**26.4**
SIFT (Sequence)	**Deleterious (0.01)**	Tolerated (0.13)	**Deleterious (0.02)**	Tolerated (1.00)	Tolerated (0.51)
PolyPhen-2 (HumVar)	**Possibly damaging (0.600)**	Benign (0.271)	**Possibly damaging (0.869)**	Benign (0.014)	**Probably damaging (1.000)**
MutationTaster	**Deleterious**	**Deleterious**	**Deleterious**	Benign	Benign
gnomAD Total	N/A	N/A	0.000001239	N/A	N/A
NCBN Total	N/A	N/A	N/A	N/A	N/A
ACMG criteria	Uncertain significance (PM1, PM2, PP3)	Uncertain significance (PM1, PM2)	Uncertain significance (PP3)	Uncertain significance (PM2, BP4)	Uncertain significance (PM2)
Clinical features					
Sex	Female	Female	Male	Female	Male
The age at disease onset (years)	24	26	37	65	57
The age at last visit (years)	36	47	55	77	83
Family history	(-)	Mother, sibling, maternal uncle, maternal grandmother, and maternal cousins	Mother, sibling, maternal uncle, and maternal aunt	Mother and sibling	Mother
Time from disease onset to wheelchair use (years)	Walking independently	Cane-assisted gait	16	12	12
Eye movements	Saccadic	Saccadic	Saccadic and upgaze pulsy	Saccadic	Saccadic
Nystagmus	Horizontal	Gaze directional	Lateral gaze directional	(−)	(−)
Dysarthria	**(**+**)**	**(+)**	**(+)**	**(+)**	**(+)**
Limb ataxia	**(**+**)**	**(+)**	**(+)**	**(+)**	**(+)**
Trunkal ataxia	**(**+**)**	**(+)**	**(+)**	**(+)**	(−)
Spasticity	**(**+**)**	(−)	(−)	(−)	**(**+**)**
Deep tendon reflex	Increase	Decrease (Lower limbs)	Increase	Decrease	Increase (Lower limbs)
Extrapyramidal signs	(−)	Postual/Intentional tremor and rigidity	(−)	No-no head tremor	(−)
Sensory disturbances	(-)	(−)	(−)	(−)	(−)
Bladder and rectal disturbances	(−)	(−)	**(**+**)**	(−)	**(**+**)**
Cognitive decline	(−)	(−)	(−)	**(**+**)**	(−)
Skin changes	**(**+**)**	(−)	(−)	(−)	(−)
Head imaging findings					
Brain stem atrophy	**(**+**)**	**(+)**	**(+)**	**(+)**	(−)
Cerebellar atrophy	**(**+**)**	**(+)**	**(+)**	**(+)**	**(+)**
White matter lesion	(−)	(−)	(−)	**(**+**)**	(−)
Other features	Hot cross bun sign	Calcification in the globus pallidus and dentate nucleus	N.P.	N.P.	N.P.

(−) absent, (+) present, *N/A* not available, *N.P.* nothing particular, *CADD* combined annotation dependent depletion, *SIFT* sorting intolerance from tolerance, *ACMG* American College of Medical Genetics, *NCBN* National Center Biobank Network

Findings indicating variant pathogenicity are shown in bold

#### ELOVL4

**Fig. 1:**
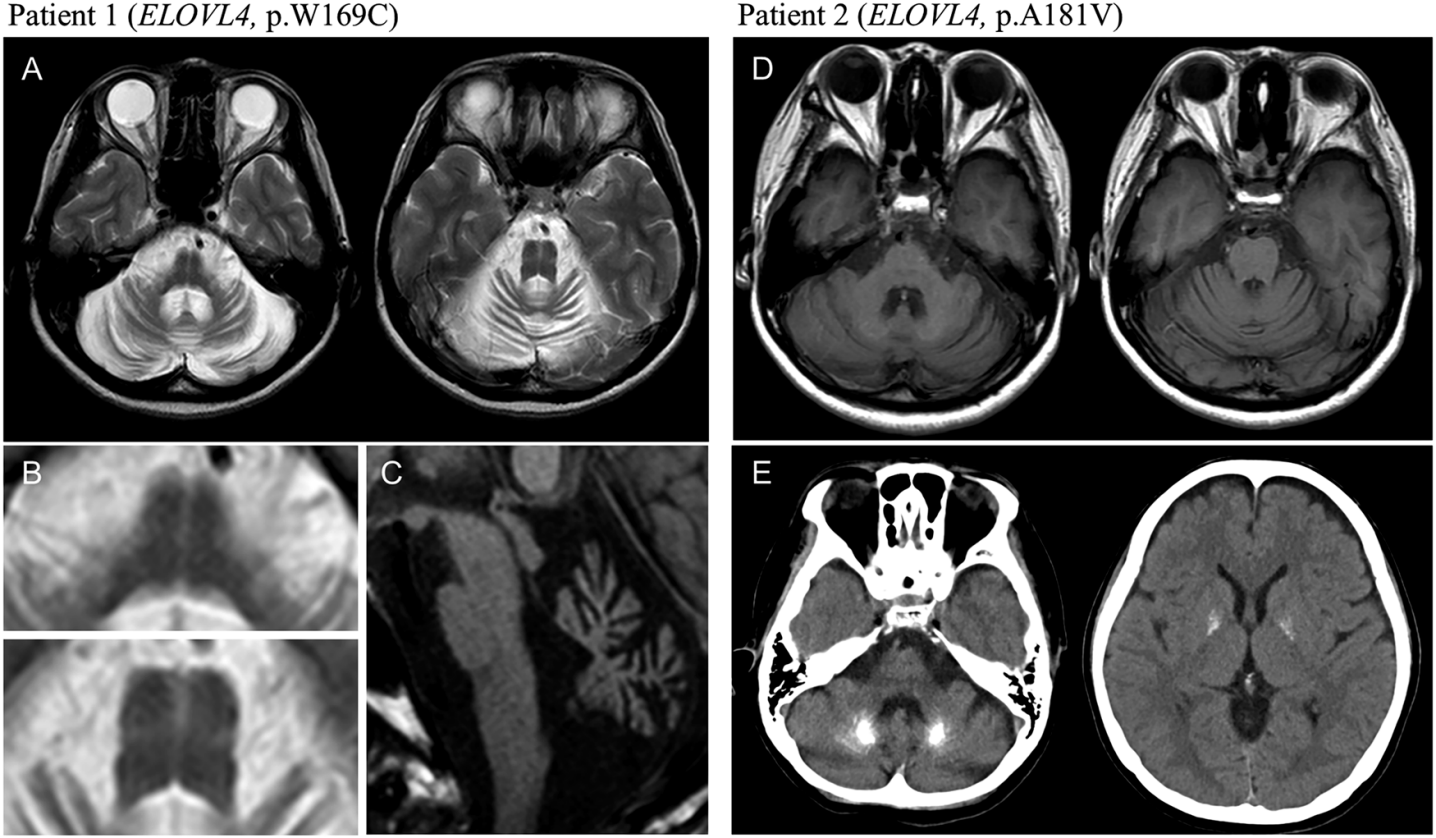
Head images of the patients with *ELOVL4* variants. **A** Axial T2-weighted images of Patient 1 show cerebellar and brainstem atrophy as well as the hot cross bun sign in the pons. **B** The hot cross bun sign is clearly observed in the enlarged images of the pons in (**A**). **C** Sagittal T1-weighted image of Patient 1 showing the cerebellar vermis and brainstem atrophy. **D** Axial T1-weighted images of Patient 2, showing mild cerebellar and brainstem atrophy. **E** Head computed tomography of Patient 2 showing calcifications in the globus pallidus and dentate nucleus

Patients 1 and 2 harbored a missense variant of *ELOVL4* (Patient 1: c.507G > C,p.W169C; Patient 2: c.542C > T,p.A181V). *ELOVL4* (elongation of very long-chain fatty acid-like 4; MIM 605512) was associated with SCA34 (MIM 133190). Skin changes, which have been previously reported as a characteristic of SCA with *ELOVL4* variants [6, 7], were observed in Patient 1 but not in Patient 2. Magnetic resonance imaging (MRI) of the head showed cerebellar and brainstem atrophy (Fig. 1A, C, D). T2-weighted images (T2WI) of Patient 1 showed the hot cross bun sign, which is characteristic of multiple system atrophy (MSA) (Fig. 1A, B). Patient 1 had no family history of the disease. Her parents were asymptomatic, but their genetic tests have not been conducted. The *ELOVL4* variant was predicted to be pathogenic by all five predictive algorithms. Calcification in the globus pallidus and dentate nucleus was observed on the head CT scan of patient 2 (Fig. 1E). Her mother, who was suspected of developing the disease without genetic testing, did not show any calcification in her brain. Patient 2 exhibited mild Parkinsonism, such as postural and intentional tremors and rigidity.

#### ELOVL5

Patient 3 harbored a missense variant in *ELOVL5* (c.179C > T, p.S60F). *ELOVL5* (elongation of very long-chain fatty acid-like 5; MIM 611805) was associated with SCA38 (MIM 615957). The patients exhibited slowly progressive SCA with limb ataxia, truncal ataxia, dysarthria, saccadic eye movement, and bladder and rectal disturbances. His head MRI data were lost and cannot be presented, but cerebellar and brainstem atrophy was confirmed from the medical record entries.

#### GRM1

**Fig. 2:**
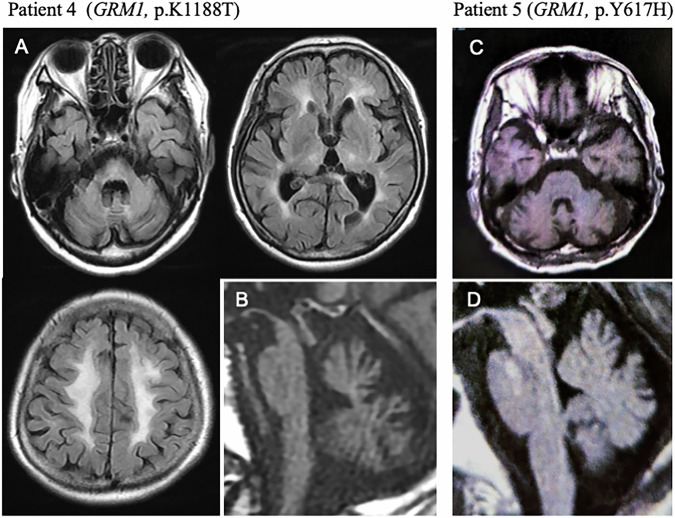
Head images of the patients with GRM1 variants. **A** Axial fluid-attenuated inversion recovery images of Patient 4 show cerebellar and brainstem atrophy and white matter lesions in the periventricular and subcortical regions. **B** Sagittal T1-weighted image of Patient 5, showing cerebellar and brainstem atrophy. Axial (**C**) and sagittal (**D**) T1-weighted images of Patient 5 showed cerebellar and brainstem atrophy

Patients 4 and 5 harbored missense variants in *GRM1* (patient 4: c.3563A > C,p.K1188T; patient 5: c.1849T > C,p.Y617H). *GRM1* (glutamate receptor metabotropic 1; MIM 604473) is associated with SCA44 (MIM 617691). The patients exhibited slowly progressive SCA with limb ataxia, saccadic eye movement, and dysarthria. Patient 4 presented with truncal ataxia, no-no head tremor, or cognitive decline (the mini-mental state examination: 15). Magnetic resonance imaging (MRI) of the patient’s head showed severe white matter lesions (Fig. 2A) and atrophy of the cerebellum and brainstem (Fig. 2A, B). Patient 5 exhibited spasticity and cerebellar atrophy (Fig. 2C, D).

## Discussion

Using WES, we identified five candidate pathogenic variants in these three genes. Previously reported cases of *ELOVL4* SCA presented with skin changes in childhood [6, 7]; however, Patient 2 did not exhibit any skin abnormalities. Notably, a previously reported Japanese family with an *ELOVL4* SCA variant also did not exhibit skin changes [8], suggesting that Japanese patients are less likely to develop skin lesions than European patients. The hot cross bun sign was observed in Patient 1 as well as in a Japanese *ELOVL4* SCA family [8]. As the hot cross bun sign can also be observed in MSA and several SCAs (including SCA1, 2, 3, 6, 7, and 8) [9], careful differential diagnosis is essential. Patient 2 exhibited mild Parkinsonism and calcification in the globus pallidus and dentate nucleus, which have not been previously reported. Since her suspected mother did not show any calcification in her brain, this may not be a characteristic feature of this variant. Japanese patients with *ELOVL4* variants have been reported to show autonomic disturbances, which were not observed in our *ELOVL4* cases. According to the American College of Medical Genetics (ACMG) criteria [10], both two patients were classified as Uncertain significance. Skin changes and the hot cross bun sign observed in Patient 1 are considered possible findings, however, whether autonomic disturbances and calcification in the brain observed in Patient 2 are characteristics of *ELOVL4* SCA in Japanese patients remains to be clarified and requires further investigation requires further investigation.

The present case of an *ELOVL5* variant exhibited bladder and rectal disturbances, but previous reports have not mentioned these symptoms [11]. According to the ACMG criteria, this patient was classified as Uncertain significance. Whether autonomic disturbances are characteristics of *ELOVL5* SCA in Japanese patients remains to be seen.

Patients with *GRM1* variants exhibited few common features, other than cerebellar symptoms. One patient showed white matter lesions, cognitive decline, and no-no head tremors, whereas the other showed spasticity. Some previous *GRM1*-related SCA exhibited spasticity [12], but none reported cognitive decline. The cognitive decline observed in our patient may have been related to white matter lesions associated with atherosclerosis because this patient had dyslipidaemia and hypertension. Both variants were classified as Uncertain significance based on the ACMG criteria. We need some other cases to determine clinical significance.

The diagnostic yield in this study was lower than that reported in the previous studies of genetic screening for autosomal dominant ataxia. Those studies reported the diagnostic yield was 14.3% [13] and 32% [14], respectively, whereas it was 2.9% in our cohort. The discrepancy is likely attributable to differences in the ethnic backgrounds of study participants and the target genes analyzed.

This study has several limitations. First, most cases could not be segregated because of the lack of family testing. Second, a functional analysis of the variant proteins was not performed. Finally, it should be noted that SCA27B, which is an important subtype in Japanese patients with SCA, was not excluded in this study. It is crucial to accumulate additional data from other families with SCA in the future.

We screened for SCA cases and identified five candidate pathogenic variants in three genes. We hope that the present findings will be valuable in future clinical practice regarding SCA.
